# Effects of D-amino acid oxidase inhibition on memory performance and long-term potentiation in vivo

**DOI:** 10.1002/prp2.7

**Published:** 2013-10-01

**Authors:** Seth C Hopkins, Una C Campbell, Michele L R Heffernan, Kerry L Spear, Ross D Jeggo, David C Spanswick, Mark A Varney, Thomas H Large

**Affiliations:** 1 Sunovion Pharmaceuticals IncMarlborough, Massachusetts; 2 Neurosolutions Ltd.Coventry, U.K; 3 Department of Physiology, Monash UniversityClayton, Victoria, Australia; 4 Warwick Medical School, University of WarwickCoventry, U.K

**Keywords:** Cognition, d-amino acid oxidase, d-cycloserine, d-Serine, Long-term potentiation, NMDA Receptors

## Abstract

*N*-methyl-d-aspartate receptor (NMDAR) activation can initiate changes in synaptic strength, evident as long-term potentiation (LTP), and is a key molecular correlate of memory formation. Inhibition of d-amino acid oxidase (DAAO) may increase NMDAR activity by regulating d-serine concentrations, but which neuronal and behavioral effects are influenced by DAAO inhibition remain elusive. In anesthetized rats, extracellular field excitatory postsynaptic potentials (fEPSPs) were recorded before and after a theta frequency burst stimulation (TBS) of the Schaffer collateral pathway of the CA1 region in the hippocampus. Memory performance was assessed after training with tests of contextual fear conditioning (FC, mice) and novel object recognition (NOR, rats). Oral administration of 3, 10, and 30 mg/kg 4H-furo[3,2-b]pyrrole-5-carboxylic acid (SUN) produced dose-related and steady increases of cerebellum d-serine in rats and mice, indicative of lasting inhibition of central DAAO. SUN administered 2 h prior to training improved contextual fear conditioning in mice and novel object recognition memory in rats when tested 24 h after training. In anesthetized rats, LTP was established proportional to the number of TBS trains. d-cycloserine (DCS) was used to identify a submaximal level of LTP (5× TBS) that responded to NMDA receptor activation; SUN administered at 10 mg/kg 3–4 h prior to testing similarly increased in vivo LTP levels compared to vehicle control animals. Interestingly, in vivo administration of DCS also increased brain d-serine concentrations. These results indicate that DAAO inhibition increased NMDAR-related synaptic plasticity during phases of post training memory consolidation to improve memory performance in hippocampal-dependent behavioral tests.

## Introduction

*N*-methyl-d-aspartate receptor (NMDAR) activation initiates a cascade of signaling events that can lead to increases in synaptic strength. NMDA receptors play a unique role in our understanding of associative memory formation due to their biophysical requirements for activation. The multiple requirements of occupancy by two agonists (glutamate on NR2 subunits, d-serine or glycine on NR1 subunits) plus membrane depolarization to relieve Mg2+ block of the channel pore, tightly couple the coincidence of multiple extracellular signals to NMDA receptor-mediated intracellular signaling responses.

One strategy to effect synaptic strength toward a therapeutic goal of cognitive improvement might be to increase the occupancy of NMDA receptor glycine sites (Yang and Svensson 2008). For example, d-cycloserine (DCS) is an exogenous agonist for this site that has been studied clinically in conditions like Alzheimer's disease, schizophrenia, and anxiety disorders (Schwartz et al. 1996; Tsai et al. 1999; Duncan et al. 2004; Goff et al. 2005; Otto et al. 2010; Litz et al. 2012). Inhibition of glycine reuptake can increase concentrations of glycine local to this site (Depoortere et al. 2005), and is being developed for the treatment of schizophrenia (Hopkins 2011). Inhibition of d-serine metabolism by d-amino acid oxidase (DAAO) may also increase concentrations of d-serine local to these sites, with effects expected to predominate where these sites are not already occupied by endogenous levels of d-serine or glycine (Papouin et al. 2012). The level of tissue d-serine is indeed already elevated in brain regions where DAAO activity is low (Schell 2004), and it has been postulated that acute DAAO inhibition is insufficient to increase d-serine to the levels required to exert therapeutic-like effects in animal models (Smith et al. 2009; Ferraris and Tsukamoto 2011). The extent to which DAAO regulation of d-serine concentrations can affect NMDAR function remains elusive.

Here we evaluate the contribution of DAAO inhibition on cognitive performance using 4H-furo[3,2-b]pyrrole-5-carboxylic acid (SUN), a compound capable of producing lasting brain inhibition of DAAO activity in rodents (Dorsey et al. 2008; Sparey et al. 2008; Duplantier et al. 2009; Ferraris and Tsukamoto 2011). We tested SUN in mice and rats for increased memory performance under experimental conditions optimized to detect performance increases above vehicle-treated animals. We observed these effects under pharmacodynamic conditions of DAAO inhibition in the hours that followed training. Furthermore, we detected an effect of DAAO inhibition on long-term potentiation in the hippocampus that was similar to that established with the NMDA receptor agonist DCS.

## Materials and Methods

### Contextual fear conditioning

SUN was evaluated for its effect on memory using the contextual fear conditioning test in male C57Bl/6 mice. To assess contextual conditioning, a standardized contextual fear conditioning task was used. Specifically, on the training day, the mouse was placed into the conditioning chamber (Med Associates, Inc., St Albans, VT) for 2 min before the onset of the unconditioned stimulus (US), a 0.75 mA foot shock of 2 sec duration. The US was repeated twice with a 1 min intertrial interval between shocks. Training was performed using an automated software package (Med Associates, Inc.). After the last training trial, a mouse was left in the conditioning chamber for another 30 sec and then placed back in its home cage. Contextual memory was tested 24 h after training. The mouse was placed into the same training chamber and conditioning was assessed by scoring freezing behavior. This behavior, a well-established measure for learned fear, was defined as complete lack of movement for more than 2½ sec of a given 5 sec, with scoring through the session broken down into 5-sec bins. The animals remained in the conditioning chamber for a total testing time of 3 min, with freezing behavior scored throughout. After each experimental subject, the experimental apparatus was thoroughly cleaned with 75% ethanol, water, and then dried, and ventilated for a few minutes. Test compounds were administered prior to training. Following training, freezing was measured every 5 sec for 30 sec, and again for 3 min during the testing period. SUN was administered 3, 10, and 30 mg/kg, orally, 2 h before the training session and compared with a vehicle control group. As a positive control, rolipram, (0.1 mg/kg, i.p.) was administered 20 min before the training session. Ten mice were tested in each group. The test was performed by investigators blinded to treatment. Each experimental condition was replicated two independent times and replicate days were added to generate final numbers of subjects.

### Novel object recognition

SUN was evaluated for its effect on memory using the novel object recognition task in male Wistar rats. The novel object recognition model is based on the greater spontaneous exploration of a novel over familiar object shown by rodents. Rats were assessed for memory performance in a test apparatus comprised of an open-field arena placed in a sound-attenuated room under dimmed lighting. Images of the open field were captured using digital camera, and viewed on a monitor in an adjoining room. Each rat was subjected to the procedure separately and care was taken to remove any olfactory/taste cues by cleaning the arena and test objects with alcohol between trials and between rats. Following a 5-min habituation period, each rat was placed into the test arena in the presence of two identical objects (plastic shapes). Each rat was placed facing the same direction at the same position in the arena, and the time spent actively exploring the objects during a 5-min test period (T1) was recorded. The rat was returned to its home cage between tests. After 24 h, each rat was again placed in the test arena for 5 min (T2) in the presence of one of the familiar objects and a novel object, and the time spent exploring both objects was again recorded. The presentation order and position of the objects (left/right) was randomized between rats to prevent bias from order or place preference. A preference index for each object, the ratio of time spent exploring both objects (during retention session T2), was used to measure memory performance.

SUN was examined at 3, 10, and 30 mg/kg, administered orally, 2 h before the training session and compared with the vehicle control group. Galantamine (3 mg/kg, i.p.) administered 60 min before the training session, was used as a positive control. Eight rats were tested in each group. The test and video analysis were performed by investigators blinded to treatment.

### Data analysis, compounds, and animals

Cognition testing was analyzed by analysis of variance (ANOVA) followed by Fisher PLSD post hoc analysis. An effect was considered significant if *P* < 0.05. d-serine dose-response curves were fit with nonlinear regression routines using GraphPad Prism 5.0. Rolipram and Galantamine was purchased from Sigma (Sigma-Aldrich, St. Louis, MO), and SUN was synthesized according to methods described in (Heffernan et al. 2009). The behavioral tests were conducted according to established protocols approved by the IACUC Committee at PsychoGenics, Inc., and in accordance with the Guide to Care and Use of Laboratory Animals (National Institutes of Health, 1996).

### Electrophysiology

The effects of DCS and SUN on the levels of long-term potentiation (LTP) recorded from the CA1 region of the rat hippocampus were evaluated in vivo. Male Sprague-Dawley rats (270–400 g) were anesthetized with an intraperitoneal injection of urethane (1 mL/100 g, 12% solution) and supplemented as necessary (0.2 mL/100 g), dependent upon the response to a paw pinch and the stability of monitored cardiovascular variables. Core body temperature was monitored and maintained at 37°C via a homoeothermic blanket and rectal probe (Harvard, Edenbridge, U.K.). The right femoral vein was cannulated for the administration of supplemental anesthetic. The right femoral artery was also cannulated, for recording of arterial blood pressure via a pressure amplifier (Neurolog Pressure Amp. NL108, Neurolog, Digitimer, Welwyn Garden City, U.K.). Exposure of the trachea, posterior to the larynx, allowed for its cannulation before placement of animals in a stereotaxic frame (Narishige ST-7, Narishige, London, U.K.). Animals were allowed to breathe room air, which in some cases was oxygen enriched. The dorsal brain surface overlying the hippocampus was exposed by removal of the overlying skin and connective tissue and subsequent removal of the skull bone. Small needle electrodes were then placed in opposing paws which, via a Neurolog head stage (model NL105) connected to a Neurolog amplifier (×10 000; Neurolog AC preamplifier NL104) and filter (0.5–5 kHz; Neurolog filter, model NL125), allowed for the recording of electrocardiogram (ECG) activity and the subsequent derivation of heart rate. The dural and pial layers overlying the brain were subsequently removed. A concentric bipolar stimulating electrode (FHC, Bowdoin, ME) and carbon fiber recording electrode (Kation Scientific, Szeged, Hungary) were then lowered through the cortex to the stratum radiatum of the CA1 region of the hippocampus according to the following stereotaxic coordinates (Paxinos and Watson 1998) for the recording electrode (Bregma −4.4, lateral 2.0–2.25, depth 2.0–2.7 mm), and the stimulation electrode (Bregma −3.4, lateral 2.5, depth 2–3 mm).

Electrical stimulation (0.1 ms pulse width, 10–100 V, 0.14 Hz) of the Schaffer collateral pathway evoked field excitatory postsynaptic potentials (fEPSPs) that were recorded using a Neurolog head stage (model NL105), connected to a Neurolog amplifier (×10 000; Neurolog AC preamplifier NL104) and filter (0.5–5 kHz; Neurolog filter, model NL125), with the subsequent output being transmitted to a PC via a micro 1401 interface (CED, Cambridge, U.K.). Cellular activity was analyzed using Spike2 software (CED), with the amplitude of the electrically evoked (0.1 ms pulse width, 10–100 V, 0.14 Hz) fEPSP presented real-time using Spike 2 online analysis. After optimization of the fEPSP by altering the depth of both stimulation and recording electrodes in 10 μm increments, an input–output curve was carried out to determine the maximal amplitude and the voltage required to generate an fEPSP of 30–50% of maximum for baseline generation. Stimulation parameters were then maintained at this level at a frequency of 0.03 Hz to demonstrate stable responses for a period of at least 10 min before commencing the full experiment protocol.

A minimum 20 min baseline period was recorded followed by theta frequency burst stimulation (TBS) of the Schaffer collateral pathway. For the purposes of establishing a full range of TBS-evoked LTP, four paradigms were selected; 2 trains of stimulation (2× TBS), 5 trains of stimulation (5× TBS), 10 trains of stimulation (10× TBS) and 20 trains of stimulation (20× TBS). Each train of stimulation consisted of a burst of four pulses at 100 Hz, each burst occurring at a (near theta) frequency of 5 Hz.

A single dose of DCS (100 mg/kg) was administered via intra-peritoneal injection 50 min prior to running a TBS protocol. A single dose of 10 mg/kg SUN was administered orally using a 16G gavage needle between 3 and 4 h prior to TBS. For each test article, a day-matched control experiment was performed on each experimental day.

### PK/PD analysis

Exposure of SUN, DCS, and d-serine concentrations were measured by liquid chromatography/tandem mass spectrometry (LC/MS/MS). d-serine concentrations in the cerebellum were monitored to observe increases over baseline following administration of SUN. Rats or mice were administered single oral doses and sacrificed at different time points following compound administration. A minimum of *n* = 3 animals were used per time point. Cerebellum tissue was collected, processed, and analyzed for compound and d-serine concentrations. Whole blood samples were collected in tubes containing potassium ethylenediaminetetraacetic acid. These tubes were centrifuged and the plasma was decanted. Plasma and cerebellum samples were stored at −80°C until analysis by LC/MS/MS for compound and d-serine concentrations.

## Results

**Figure 1:**
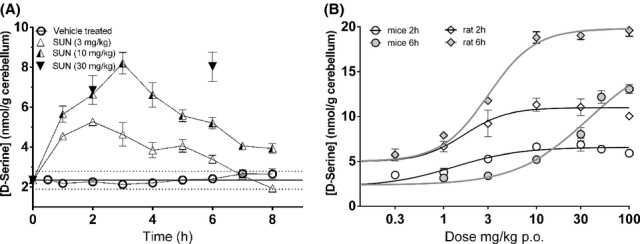
(A) Time courses of cerebellar tissue d-serine concentration in response to administration of SUN 3, 10, 30 mg/kg, p.o. to mice. Solid horizontal line represents the vehicle-treated mean d-serine concentration ± 1 standard deviation (*N* = 85). (B) Dose-response relationship of SUN on cerebellar tissue d-serine concentrations resulting 2 and 6 h after administration of SUN p.o. in mice (circles) and rats (diamonds), where solid lines are nonlinear regression curves to interpolate half-maximal d-serine increases. Data are mean ± SEM, with between 4 and 7 animals each.

The pharmacodynamics of d-serine, in response to these doses of SUN, was examined in mice and rats following systemic administration (Figure 1). d-Serine concentrations in cerebellum tissues were used to interpret the extent of DAAO inhibition, because of the low baseline d-serine concentrations relative to other brain areas. In mice, baseline d-serine concentrations in forebrain tissue (the content of the entire brain after dissecting away brainstem and cerebellum tissue) were 157 ± 13 nmol/g (mean ± SD, *N* = 9). In contrast, baseline cerebellum concentrations were 2.35 ± 0.45 nmol/g (*N* = 85). d-Serine concentrations in cerebellum tissue increased promptly following SUN administration (Figure 1A). d-Serine increases following 3 mg/kg p.o. SUN peaked around 2 h, and declined toward baseline during the following 4 h. The higher doses of 10 and 30 mg/kg p.o. produced a maximal rate of increase in d-serine for at least 2 h. Peak d-serine increases following 10 mg/kg SUN occurred at 3 h, 1 h later than the 3 mg/kg dose. The highest dose tested (30 mg/kg) resulted in higher d-serine concentrations at the 6 h time point, but not at the 2 h time point, indicating that any dose-dependent increase in the rate of d-serine increase was saturated during the first 2 h after administration (100% inhibition of DAAO). Overall, the time to peak d-serine concentration was dose-dependent, as was the consequential decline back to baseline. To understand this dose-dependence, increasing doses of SUN were administered to both rats and mice, and d-serine concentrations were measured at 2 and 6 h (Figure 1B). Likewise, the baseline d-serine concentration in rat cerebellum tissue (4.97 ± 1.45 nmol/g, *N* = 88) was lower than forebrain areas (175 ± 19 nmol/g, *N* = 59). Increasing doses of SUN also produced a saturating level of d-serine at 2 h and also at 6 h, indicative of a constant rate of increase during periods of maximal DAAO inhibition. In rats, the dose for half-maximal response in d-serine was 1.6 mg/kg (1.0, 2.7) at 2 h, and 3.0 mg/kg (2.6, 3.5) at 6 h (95% confidence intervals from nonlinear regression). In mice, the dose for half-maximal response in d-serine was 1.3 mg/kg (0.86, 1.9) at 2 h, and 36 mg/kg (22, 58) at 6 h. Taken together, these results indicate a similar half-maximal dose for a maximal d-serine response between the two species when estimated at 2 h, but the effects of SUN on d-serine increases appeared to last longer in rats than those in mice.

**Figure 2:**
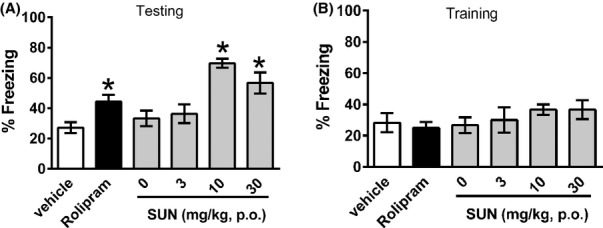
(A) Effect of SUN on contextual memory in mice tested 24 h after training. Freezing behaviors were scored over the 3-min test and represent mean ± SEM. (B) Effect of SUN on immediate freezing responses measured 30 sec after training (to control for acute effects of treatments on freezing behaviors). *P < 0.05 by one-way ANOVA. Rolipram-positive control (0.1 mg/kg i.p.) was administered 20 min before training session, and compared to its vehicle control (1% DMSO). SUN was administered (3, 10, 30 mg/kg, p.o.) 2 h before training, and compared to its vehicle control (50 mmol\L isotonic phosphate buffer, 0 mg/kg in figure). *N* = 10 animals per group.

On the basis of the pharmacodynamic/pharmacokinetic (PK/PD) relationship for SUN observed in both mice and rats, we looked for effects of DAAO inhibition on memory. Oral administration of SUN increased contextual memory performance contextual fear conditioning. Mice administered 10 and 30 mg/kg SUN 2 h prior to training and assessed 24 h after training froze significantly more than vehicle-injected mice (Figure 2A). Similarly, mice administered rolipram as a positive control, froze significantly more than their corresponding vehicle-injected mice. Importantly, there were no effects of either compound on immediate freezing responses measured 30 sec after training (Figure 2B), indicating that the observed increase in freezing after 24 h did not simply reflect greater pain sensitivity or baseline anxiety.

**Figure 3:**
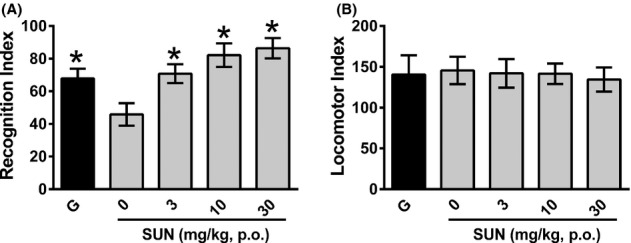
(A) Effect of SUN on novel object recognition memory in rats tested 24 h following training. Recognition index is the% time spent exploring the novel object out of the total time spent exploring both objects, scored over 5 min. Data are mean ± SEM. (B) Effect of SUN on exploratory behaviors during a 5 min habituation period immediately prior to training (to control for acute effects of treatments on exploratory and locomotor behaviors). *P < 0.05 by one-way ANOVA. Galantamine-positive control (3 mg/kg i.p.) was administered 60 min before training session. SUN was administered (3, 10, 30 mg/kg, p.o.) 2 h before training, and compared to its vehicle control (50 mmol\L isotonic phosphate buffer, 0 mg/kg in figure). *N* = 8 animals per group.

The effects of SUN on rat novel object recognition assessed 24 h following training are shown in Figure 3. In comparison to vehicle-treated rats, both galantamine and SUN (at all doses) increased recognition performance. During the initial 5-min acclimatization period in the apparatus, animals were scored for exploratory behavior. No significant difference in locomotion was observed between any of the treatment groups, indicating effects were not a result of changes in exploratory behaviors as assessed in the apparatus immediately prior to testing.

**Figure 4:**
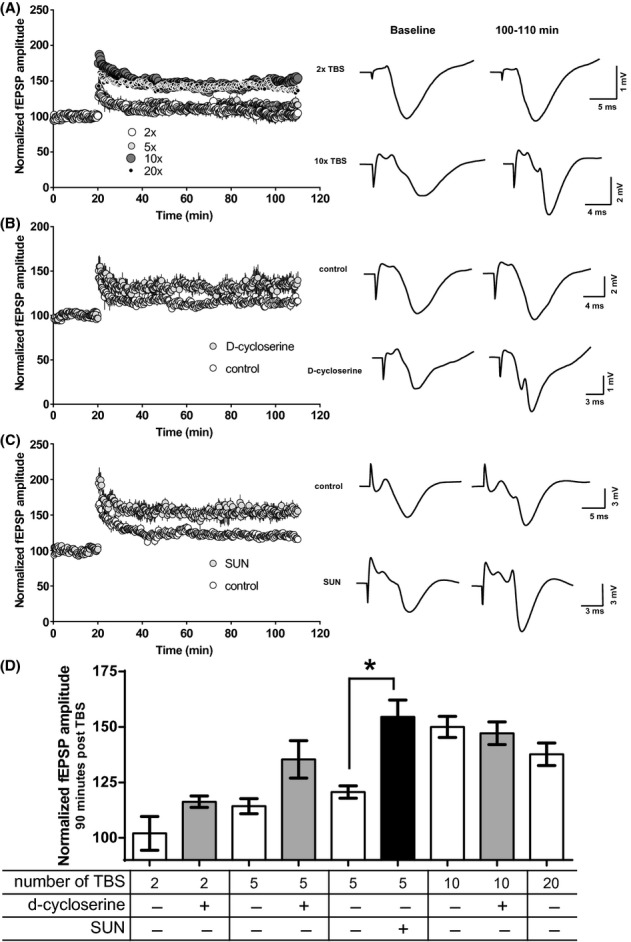
(A) Effects of increasing the number of trains of theta burst stimulus (2× TBS, 5× TBS, 10× TBS, and 20× TBS) on in vivo LTP in anesthetized rats. Representative fEPSP traces are shown. (B) Effect of 100 mg/kg i.p. DCS administered 50 min prior to 5× TBS, *N* = 5 rats each group. (C) Effect of 10 mg/kg SUN p.o. 3–4 h prior to 5× TBS, with day-matched parallel vehicle control groups of *N* = 8 rats each treatment group. (D) Increase above baseline (100%) LTP for all treatment groups and stimulus intensities, measured at 80–90 min post-TBS, and represented as mean ± SEM. *P < 0.0001 by one-way ANOVA.

To determine if these effects of SUN on memory performance also corresponded to an effect of DAAO inhibition on changes in hippocampal synaptic plasticity, we developed an in vivo LTP paradigm sensitive to increases in NMDA receptor function. In urethane-anesthetized rats, fEPSPs recorded from the CA1 region of the hippocampus were potentiated above baseline following a train of TBS stimuli, and increasing the number of TBS bursts produced a corresponding increase in LTP (Figure 4A). The effects of 10× TBS were to increase LTP to 150 ± 4.7% (mean ± SEM) of baseline and increasing the bursts to 20× TBS did not further increase LTP (138 ± 5.1%). DCS administration (100 mg/kg i.p. 50 min prior to TBS) increased the level of LTP following the submaximal 5× TBS (Figure 4B), but not following the 2× TBS or the maximal 10× TBS stimuli (Figure 4D). Therefore, the submaximal 5× TBS paradigm was used to evaluate the effects of SUN when administered p.o. 3–4 h prior to testing for LTP. Here 10 mg/kg p.o. SUN increased the level of LTP (Figure 4C) that was statistically significant out to 80–90 min post stimulus. Figure 4D summarizes the responses to treatment conditions and across stimulus intensities, as quantified at 80–90 min post stimulus.

**Figure 5:**
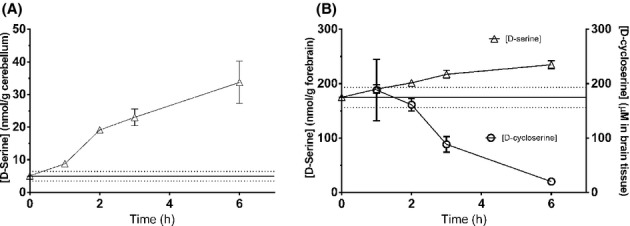
DCS increased d-serine concentrations in cerebellum (left panel) and forebrain (right panel) following administration of 125 mg/kg i.p. to rats. The concentrations of DCS in brain tissue (circles in right panel) declined over the course of 6 h. Error bars in figure are SEM, with between 3 and 7 animals each. Baseline d-serine concentrations ± SD are represented by lines ± dotted lines in figures.

Finally, we report the brain concentrations of DCS following 125 mg/kg i.p. administration. DCS was measured by LC/MS/MS in a group of rats administered 125 mg/kg i.p. DCS. DCS was observed at ∼200 μmol\L in brain tissue (Figure 5, assuming 1 g tissue approximates 1 mL volume). Interestingly, DCS administration caused a steady and large increase in d-serine concentrations in both cerebellum tissue and forebrain tissue, corresponding to what has been reported for extracellular concentrations by microdialysis (Fujihira et al. 2007). This increase is apparent above the elevated mean baseline d-serine concentrations of forebrain tissue. The increases following DCS administration were above 1 SD of baseline after 2 h post administration.

## Discussion

Here we report on the pharmacodynamic requirements for DAAO inhibition to enhance memory performance in rodents. By addressing NMDAR hypofunction, DAAO is a therapeutic target for the treatment of schizophrenia, especially for improving cognitive and negative symptoms. These results provide pharmacological support in rodents for a role of DAAO activity in hippocampal NMDA receptor function, synaptic plasticity, and memory consolidation.

The doses of SUN used in this study achieved full inhibition of central DAAO activity. SUN administration caused prompt and steady increases in d-serine concentrations above the relatively low baseline in cerebellum tissue. The ∼30-fold higher baseline d-serine concentrations between forebrain regions and the cerebellum in this study inversely related to the difference in the levels of DAAO enzyme between these regions (Horiike et al. 1994; Nagata et al. 1994; Morikawa et al. 2001; Rais et al. 2012). Indeed, d-serine concentrations likely also increase in brain areas outside of the cerebellum, although an increase as large as the 10–20 nmol/g seen in cerebellum would still be within one standard deviation of forebrain d-serine baseline values. Bulk tissue d-serine concentrations may not reflect local synaptic concentrations proximal to sites of action, and d-serine or glycine effects may be restricted to synaptic and extrasynaptic NMDARs, respectively (Papouin et al. 2012). If DAAO activity were proximal to NMDA receptors and responsible for maintaining glycine sites unoccupied, exogenous d-serine would not persist long in areas of elevated DAAO activity. The effects of DAAO inhibition on memory performance and hippocampal LTP support the role for DAAO regulating d-serine and NMDAR-induced plasticity at hippocampal synapses.

d-Serine concentrations provided a useful pharmacodynamic indicator of DAAO inhibition, with the assumption that tissue d-serine production was balanced by DAAO activity. The 3 mg/kg p.o. dose in mice reached peak d-serine levels after 2 h, followed by a return to baseline levels by 6 h (Figure 1B). Under our assumption, DAAO activity was being re-established after 2 h. The ineffective 3 mg/kg dose in mice suggests that DAAO inhibition post training, and not simply during acquisition, was required for the observed effects on memory. d-serine concentrations were still elevated 6 h following the 10 and 30 mg/kg p.o. doses, indicating DAAO inhibition continued 4 h post training sufficient for cognitive effects. This interpretation is consistent with the corresponding pharmacodynamics observed in rats. Here the 2 and 6 h time points following the 3, 10 and 30 mg/kg p.o. dose represent continued increases in d-serine (6 h levels are higher than corresponding 2-h levels, Figure 2). The ongoing DAAO inhibition in rats at all three dose levels was sufficient for the cognitive effects observed and explained the improvements observed at all three dose levels in rats.

We used an in vivo paradigm of LTP to investigate the putative hippocampal site of action for the cognitive effects observed with DAAO inhibition. Both mouse contextual fear conditioning and rat novel object recognition are sensitive to impairments in hippocampal function (Chen et al. 1996; Hammond et al. 2004). NMDA receptor function plays an important role in initiating the synaptic changes leading to LTP, and its activation is required for hippocampal-dependent memory consolidation (Maren et al. 1994). The TBS paradigm was used to model the conditions where NMDAR activity might contribute to memory consolidation post training. To detect enhancements above baseline, stimulus strength was adjusted by increasing the number of TBS bursts to identify a subthreshold level. The procognitive and hippocampal effects reported here are consistent with the enhancement of hippocampal theta rhythm reported by (Strick et al. 2011) with the same DAAO inhibitor. The enhancement of LTP by DCS verified that these conditions were sensitive to the pharmacological effects at d-serine-binding sites on NMDARs. Incidentally, DCS demonstrated pronounced increases in tissue d-serine following in vivo administration, as was also reported by other investigators using similar doses (Fujihira et al. 2007; Horio et al. 2013).

In summary, the DAAO inhibition ongoing during periods of post training memory consolidation increased performance in rodent cognition models. The PK/PD relationship of DAAO inhibition and memory performance corresponded to enhancement of LTP at hippocampal synapses. These results support further investigations into the therapeutic potential of DAAO inhibitors for the improvement of memory in human diseases of impaired cognitive function.

## Abbreviations

DAAO: d-amino acid oxidase
DCS: d-cycloserine
ECG: electrocardiogram
fEPSPs: field excitatory postsynaptic potentials
LTP: long-term potentiation
NMDARs: *N*-methyl-d-aspartate receptors
SUN: 4H-furo[3,2-b]pyrrole-5-carboxylic acid
TBS: theta-burst stimulation
